# Valorisation of Blackcurrant Pomace by Extraction of Pectin-Rich Fractions: Structural Characterization and Evaluation as Multifunctional Cosmetic Ingredient

**DOI:** 10.3390/polym16192779

**Published:** 2024-09-30

**Authors:** Marija Ćorović, Anja Petrov Ivanković, Ana Milivojević, Milica Veljković, Milica Simović, Paula López-Revenga, Antonia Montilla, Francisco Javier Moreno, Dejan Bezbradica

**Affiliations:** 1 Faculty of Technology and Metallurgy, University of Belgrade, Karnegijeva 4, 11000 Belgrade, Serbia; amilivojevic@tmf.bg.ac.rs (A.M.); mcarevic@tmf.bg.ac.rs (M.S.); dbez@tmf.bg.ac.rs (D.B.); 2 Innovation Center, Faculty of Technology and Metallurgy, University of Belgrade, Karnegijeva 4, 11000 Belgrade, Serbia; apetrov@tmf.bg.ac.rs (A.P.I.); mveljkovic@tmf.bg.ac.rs (M.V.); 3 Grupo de Química y Funcionalidad de Carbohidratos y Derivados, Instituto de Investigación en Ciencias de la Alimentación, CIAL (CSIC-UAM), 28049 Madrid, Spain; p.lopez.revenga@csic.es (P.L.-R.); a.montilla@csic.es (A.M.); javier.moreno@csic.es (F.J.M.)

**Keywords:** blackcurrant pomace, sequential extraction, pectin, cosmetics, emulsifier, antioxidant, skin prebiotic

## Abstract

Blackcurrant pomace is a widely available waste stream derived from the industrial production of juice rich in pectin and unextracted polyphenols. Since pectin, an emerging class of gastrointestinal prebiotics, is also a common cosmetic ingredient, the aim of this work was to evaluate blackcurrant pomace as a source of pectin-rich fractions suitable for application in prebiotic cosmetics. Hereby, this raw material was valorised by sequential extraction of acid-soluble (by citric acid, CAP) and Ca-bound (by ammonium oxalate, AOPP) pectic polysaccharides. Both fractions had favourable physicochemical features and a similar degree of methyl-esterification between low- and high-methoxyl pectin (approx. 50%), but CAP had significantly higher galacturonic acid content (72.3%), branching, and purity. Regardless of that, both had very high oil (18.96 mL/g for CAP and 19.32 mL/g for AOPP) and water (9.97 mL/g for CAP and 7.32 mL/g for AOPP)-holding capacities and excellent emulsifying properties, making them promising cosmetic ingredients. The polyphenol content was 10 times higher in CAP, while corresponding antioxidant activity was 3-fold higher. Finally, the influence of varying CAP and AOPP concentrations on common skin pathogen, *Staphylococcus aureus*, and beneficial skin bacteria, *Staphylococcus epidermidis*, was examined. The results show significant prebiotic potential of two pectic fractions since they were capable of selectively stimulating *S. epidermidis*, while *S. aureus* growth was inhibited, whereas CAP demonstrated a particularly high capacity of up to 2.2, even with methicillin-resistant *S. aureus*.

## 1. Introduction

Human skin is covered by diverse microbial communities called skin microbiota, which play a crucial role in skin immunity, repair, and antimicrobial defence. Coagulase-negative staphylococci are among the most abundant representatives of healthy skin microbiota, whereas recent studies demonstrated their significant role in skin physiology and health [1]. Being the most studied among them, *Staphylococcus epidermidis* received the label of beneficial skin microbe and emerging skin probiotic due to its role in antimicrobial defence and its ability to promote skin immunity and repair [2]. On the other hand, coagulase-positive *Staphylococcus aureus* is the most common skin pathogen, which is present in lesions of atopic dermatitis and psoriasis patients, causing persisting infections typically treated by antibiotics. Since this type of therapy unselectively kills both pathogens and beneficial microbes of the skin, searching for alternative or supplementary therapies, called skin prebiotics, is of high relevance. Although the definition of skin prebiotics is still evolving, prospective candidates to be applied on *S. aureus*-affected skin should be substances that stimulate the growth of microbes that are producing *S. aureus*-targeting bacteriocins (such as *S. epidermidis*) and selectively inhibiting *S. aureus* growth [3].

Pectin is a complex polysaccharide found widely in the cell walls and middle lamina of higher plants, which consists primarily of galacturonic acid (GalA) residues [4]. Key structural features of pectin include extensive branching in the rhamnogalacturonan-I (RGI) domain, heterogeneous distribution of acetylated GalA residues, and the presence of neutral sugars in its side chains. In general, its structural complexity varies depending on the plant source and extraction method employed [5]. Biochemically, pectin exhibits several properties influenced by its structure, including its ability to form gels in the presence of sugars and acid pH for high-methoxy pectin, while low-methoxy pectin forms a gel in the presence of divalent cations and the pH ranges from 2 to 9.50. It can also act as an emulsifier and emulsion stabiliser and functions as a dietary fibre in human nutrition [6]. These unique structural and biochemical properties enable pectin to find diverse applications in industries such as food, cosmetics, pharmaceuticals, and the development of edible films, foams, paper substitutes, and plasticisers [7]. Due to its natural origin and proven safety, it is a common emulsifier and thickening agent in cosmetic and skin care products with moisturising and hydrating properties [8]. It forms a film and improves skin barrier function while soothing and giving anti-inflammatory and antioxidant benefits [9,10]. In addition to all these functionalities, it is known that pectin has prebiotic-like activity by promoting the growth of beneficial gastrointestinal microorganisms. However, its prebiotic effect on skin microbiota representatives has not been extensively examined [11]. It was previously shown that pectin and its oligosaccharides could promote the growth of different beneficial gut microbes such as *Bifidobacteria*, *Lactobacilli*, *Enterococcus*, *Eubacterium rectale*, *Faecalibacterium prausnitzii*, *Clostridium*, *Anaerostipes*, *Roseburia* spp., etc. [12,13,14,15]. Regarding skin microbiota representatives, it was previously shown that waste lemon peel pectin extracted by hydrodynamic cavitation in water had significant antibacterial activity against *S. aureus*, which was superior to that exhibited by commercial citrus pectin [16]. Another piece of research demonstrated a strong inhibitory effect of high molecular weight (Mw) pectin polysaccharides derived from citrus peel pectin against *S. aureus*; however, beneficial *S. epidermidis* was inhibited as well [17].

The utilisation of pectin-rich agro-industrial waste as a pectin source is a widespread approach that lowers the environmental impact of related industries and gives value-added products with diverse applicability, including skin care. Besides established pectin sources, such as sugar beet pulp, citrus peel, and apple pomace, various fruit, vegetable, and maise residues are being examined as potential sources of pectin with different chemical structures, physicochemical properties, and potential functionalities, such as prebiotics. Berry pectins are particularly attractive for skin application due to their excellent gelling potential, texture enhancement, and antioxidant properties originating from bound polyphenols [18,19]. Blackcurrant stands out as a promising source of pectin due to its high pectin content and wide availability of its pomace, which remains after juice extraction [20,21,22]. It is particularly attractive for topical application since its anthocyanin-rich extract previously demonstrated in vitro prebiotic activity against skin microbiota by showing a selective stimulatory effect on *S. epidermidis* strains while inhibiting pathogenic *S. aureus* [23].

Hereby, we applied sequential extraction of acid-soluble and Ca-bound pectin-rich fractions from blackcurrant pomace by using citric acid and ammonium oxalate as extracting agents, respectively. Without further fractionation steps, we comparatively assessed their physicochemical and structural characteristics along with techno-functional and antioxidant properties important for application in different products. Having in mind the emerging necessity for new, multifunctional cosmetic ingredients of natural origin and growing awareness of skin microbiota importance, we accessed the applicability of two pectin-rich fractions as prospective skin prebiotics. The effect on *S. aureus* and *S. epidermidis* growth as relevant inhabitants of infected and healthy skin, respectively, was examined in order to evaluate their effect on skin microbiota. Since methicillin-resistant *S. aureus* (*S. aureus* MRSA) infections became a major concern, especially in hospital environments, due to resistance of this strain to many antibiotics, it was included in this study. To the best of our knowledge, pectin or pectin-like substances were not previously examined as potential skin prebiotics, and this is the first time that sequentially extracted blackcurrant pomace pectin-rich fractions were characterised in parallel and estimated as novel, plant-derived cosmetic ingredients with skin microbiota rebalancing properties.

## 2. Materials and Methods

### 2.1. Materials

Lyophilized blackcurrant was supplied from Drenovac (Belgrade, Serbia). Citric acid, ammonium oxalate, sodium carbonate, hexamethyldisilazane, trifluoroacetic acid (TFA), pullulan standards, galacturonic acid (GalA), xylose (Xyl), arabinose (Ara), rhamnose (Rha), galactose (Gal), glucose (Glc), mannose (Man), phenyl-*β*-D-glucoside, KBr, iron(III) chloride hexahydrate, pyridine, hydroxylamine chloride 6-hydroxy-2,5,7,8-tetramethylchroman-2-carboxylic acid (Trolox), Karl Fischer reagents, bovine serum albumin (BSA), and Bradford reagent were purchased from Sigma-Aldrich (St. Louis, MO, USA). Sunflower oil was produced by Dijamant (Zrenjanin, Serbia). Ethanol (96%) was acquired from Zorka Pharma (Šabac, Serbia). Folin-Ciocalteu reagent was acquired from Carlo Erba (Arese, Italy). Gallic acid was obtained from Merck (Darmstadt, Germany). 2,4,6-tri(2-pyridyl)-s-triazine (TPTZ) was purchased from Thermo Fisher Scientific (Geel, Belgium). Bacterial strain *S. epidermidis* DSM 20044 was purchased from The Leibniz Institute DSMZ (German Collection of Microorganisms and Cell Culture GmbH, Braunschweig, Germany). *S. epidermidis* ATCC 12228 and *S. aureus* ATCC 25923 were obtained from The American Type Culture Collection (Rockville, MD, USA), while *S. aureus* MRSA was acquired from The National Institute for Public Health and the Environment (RIVM, Bilthoven, The Netherlands). All mediums for microorganism cultivation were obtained from Torlak (Belgrade, Serbia).

### 2.2. Pectin-Rich Fractions Isolation

**Figure 1:**
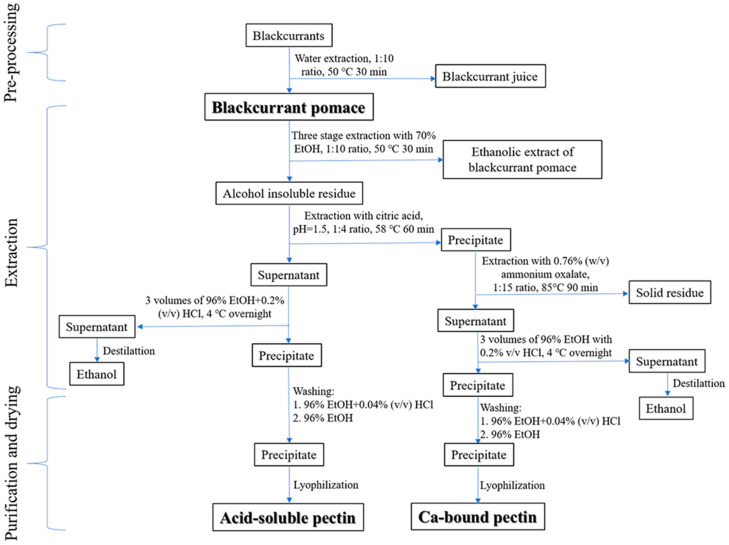
Schematic representation of pectin-rich fraction extraction procedure from blackcurrant pomace.

Powdered blackcurrants of the Ben Nevis variety (60 g dry weight) were subjected to extraction with water in a solid–liquid ratio of 1:10 at 50 °C for 60 min with constant agitation at 250 rpm (IKA KS 4000 i Control, Staufen, Germany). The extraction time and temperature were within the range of industrially applied conditions and were chosen based on previous optimisation, which provided high polyphenol recovery [23]. The obtained mixture was centrifuged using Hermle Z206-A Centrifuge (Maschinenfabrik Berthold Hermle AG, Gosheim, Germany) at 4430× *g* for 10 min, and the precipitate, blackcurrant pomace, was collected. In order to obtain the alcohol-insoluble residue (AIR), the pomace was further extracted within three consecutive stages with 70% (*v*/*v*) ethanol at a 1:10 solid-to-liquid ratio for 30 min under the same conditions. After filtration, two-stage pectin extraction was performed from the remaining pomace. The entire procedure is presented in Figure 1, while photographs of blackcurrant pomace, extract, and pectin-rich fractions are shown in Figure S1.

To obtain the acid-soluble fraction (CAP), the method previously described by Muñoz-Almagro et al. was used with minor modifications [18]. Briefly, blackcurrant pomace was re-suspended in 240 mL of 20% citric acid solution (pH = 1.5) and incubated for 60 min at 58 °C and 250 rpm. Afterwards, the precipitate was separated by centrifugation at 4430× *g* for 10 min and used for the calcium-bound fraction (AOPP) isolation. The supernatant was mixed with two volumes of 96% (*v*/*v*) ethanol with 0.2% (*v*/*v*) HCl and left overnight at 4 °C. The precipitated pectin-rich fraction was separated by centrifugation, washed first with a small amount of 0.04% (*v*/*v*) HCl in 96% (*v*/*v*) ethanol and, finally, with pure 96% (*v*/*v*) ethanol. The obtained CAP was lyophilised and used for further investigation. The calcium-bound fraction (AOPP) was obtained by mixing the aforementioned precipitate with 0.76% (*w*/*v*) ammonium oxalate in a 1:15 (*w*/*v*) ratio and incubating the mixture at 85 °C with agitation for 90 min, as previously described by Ma and co-workers [24]. The obtained supernatant was further processed as previously described in order to isolate the AOPP, which was also lyophilised and stored for future use.

### 2.3. Pectin-Rich Fractions Characterization

Extracted pectin-rich fractions were characterised by measuring their pH, water activity (a_w_), moisture, and protein content. A pH of 1% (*w*/*v*) solution of both fractions was determined using a pH meter (Mettler Toledo GmBH, Schwerzenbach, Switzerland). For the a_w_ measurement, Karl-Fischer apparatus (Mettler Toledo, Columbus, OH, USA) was used, while moisture content was determined by a KERN DBS moisture analyser (KERN & SOHN GmbH, Balingen, Germany). Protein content in pectin-rich fractions was determined by the Bradford method. Briefly, 60 μL of a sample solution in water (5 mg/mL) and 3 mL Bradford reagent were combined and allowed to react for 5 min in the dark. The absorbance was read at 595 nm, and final results were calculated based on a standard curve previously calibrated using BSA as standard and expressed as mg of proteins per g of pectin.

### 2.4. Structural Characterization

#### 2.4.1. Monomeric Composition Determination by GC-FID Analysis

To determine the monomeric composition of both pectin-rich fractions, a previously established method was employed [25]. The hydrolysis of 30 mg of the sample was performed by treating it with 1.5 mL of 2 M TFA at 110 °C under inert conditions for 4 h. Then, 300 μL of the sample was evaporated to remove the acid and 400 μL of a phenyl-*β*-D-glucoside internal standard solution (0.5 mg/mL) was added before further evaporation. For derivatization, 250 μL of 2.5% hydroxylamine chloride in pyridine was added to the residue, mixed thoroughly, and incubated at 70 °C for 30 min to form oximes. Next, 250 μL of hexamethyldisilazane (HMDS) and 25 μL of TFA were added, and the mixture was shaken and kept at 50 °C for 30 min. Afterward, the samples were centrifuged at 10,000 rpm for 2 min, and the supernatant was collected and stored at 4 °C. The gas chromatography analysis of samples with flame ionization detection (GC-FID) was performed using an Agilent Technologies 7820A Gas Chromatograph (Agilent Technologies, Wilmington, DE, USA) equipped with a VF-5HT capillary column (30 m × 0.250 mm × 0.10 μm; 5% phenylmethylpolysiloxane, 95% dimethyl-polysiloxane, Agilent J&W, Folsom, USA). The injector temperature was set to 280 °C, and the detector temperature was 385 °C. Nitrogen served as the carrier gas with a flow rate of 1 mL/min. The temperature program was as follows: an initial temperature of 120 °C with a ramp of 3 °C/min up to 270 °C. Data were obtained using Agilent ChemStation software version Rev. B.04.03 (Wilmington, DE, USA). Response factors were determined by analysing monosaccharides (glucose, mannose, rhamnose, arabinose, xylose, galactose, and galacturonic acid) in concentrations ranging from 0.02 to 2 mg/mL. Based on monosaccharide composition, different structural parameters were calculated, as previously described [25].

The homogalacturonan (HG) content was determined as

(1) HG(%)=Galacturonic acid−Rhamnose

The rhamnogalacturonan I (RGI) was determined as

(2) $$RGI\left(\%\right)=2\cdot Rhamnose+Arabinose+Galactose$$

The degree of branching of RGI (DB-RGI) backbone was determines as

(3) $$DB\text{-}RGI=\frac{Galacturonic\;acid}{Rhamnose}$$

The extent of branching of RGI (EB-RGI) backbone was determines as

(4) $$EB\text{-}RGI=\frac{Arabinose+Galactose}{Rhamnose}$$

The linearity of the pectin backbone (LP) was determined as

(5) $$LP=\frac{Galacturonic\;acid}{Rhamnose+Arabinose+Galactose}$$

The pectin purity (PP) was determined as

(6) $$PP=\frac{Galacturonic\;acid+Rhamnose+Arabinose+Galactose}{Glucose+Manose}$$

#### 2.4.2. Molecular Weight Distribution Determination by HPSEC-ELSD Analysis

The molecular weight (Mw) distribution was estimated using a previously described method [25]. For analysis, samples were first dissolved in distilled water in order to obtain the concentration of 20 mg/mL and heated at 50 °C for 30 min. The resulting solution was then mixed with a mobile phase of 0.04 M ammonium acetate in a 1:9 ratio, filtered, and analysed by High Performance Size Exclusion Chromatography with an Evaporative Light Scattering Detector (HPSEC-ELSD) from Agilent Technologies (Boblingen, Germany). The separation was performed on two TSK-Gel columns connected in series: G5000 PWXL (7.8 mm × 300 mm, 10 μm) and G2500 PWXL (7.8 mm × 300 mm, 6 μm), and a TSK-Gel guard column (6.0 mm × 400 mm) from Tosoh Bioscience (Stuttgart, Germany). The Pullulan standard set (Mw 0.342–788 kDa and concentrations 2–0.2 mg/mL), which is a frequently utilized calibration standard for aqueous size exclusion chromatography, was used to determine the Mw distribution. This standard set was previously used for determining molecular weight distribution of different carbohydrate polymers, including pectin and pectin-like substances [18,25].

#### 2.4.3. XRD Analysis

The XRD analyses of samples were conducted using an X-ray diffractometer (Bruker D8 Lynxeye XE-T, Karlsruhe, Germany) equipped with Cu Kα radiation, at 40 kV and 40 mA. The samples were scanned within the range of a 5–60° diffraction angle (2θ) with a step size of 0.02° (2θ) and a counting time of 1 s/step. These analyses were carried out by the Servicio Interdepartamental de Investigación-Universidad Autónoma (SIdI-UAM) of Madrid (Spain).

#### 2.4.4. FTIR Analysis

FTIR analysis of both extracted fractions was performed using a Bruker IFS66v (Bruker, Mineapolis, MN, USA) instrument. KBr discs were prepared by mixing the samples with KBr (1:100) pressing them. Data were collected in absorbance mode using a frequency range of 4000–400 cm^−1^ and resolution of 4 cm^−1^ (mid-infrared region) with 250 co-added scans [26]. The degree of methyl-esterification (DM) was determined as the average ratio of the peak area at 1730 cm^−1^ (COO–R) over the sum of the peak areas of 1730 cm^−1^ (COO–R) and 1640 cm^−1^ (COO^−^). 

### 2.5. Determination of Techno-Functional and Antioxidant Properties

#### 2.5.1. Water Retention Capacity

The water retention capacity (WRC) of pectin-rich fractions was examined by combining 0.1 g of the sample with 2 mL of water and incubating the mixture for 24 h with agitation at 250 rpm at 25 °C. The samples were then centrifuged at 1050× *g* for 30 min. The results were expressed as the volume of water held per gram of tested fraction.

#### 2.5.2. Oil-Holding Capacity

The oil-holding capacity (OHC) of pectin-rich fractions was determined by combining 0.1 g of the sample with 2 mL of sunflower oil and incubating the mixture for 30 min with agitation at 250 rpm at 25 °C. After that, the samples were centrifuged at 1050× *g* for 30 min and the volume of oil in the supernatant was measured. The results were expressed as the amount of oil retained per gram of the tested fraction.

#### 2.5.3. Emulsifying Properties

The emulsifying properties of the pectin-rich fractions’ emulsifying capacity (EC) and emulsion stability (ES) were determined by mixing 10 mL of sample solution (1% (*w*/*v*)) with 5 mL of sunflower oil and vigorously vortexing the mixture for 3 min to homogenize it. Subsequently, the emulsion was centrifuged at 950× *g* for 5 min. The EC was determined using the following equation, as previously shown by Bayar et al. [27]:

(7) $$EC\left(\%\right)=\frac{V_{f}}{V_{i}}\cdot 100$$

where V_f_ is the volume of emulsified layer obtained after centrifugation and V_i_ is the initial volume of the mixture.

To evaluate the emulsion stability, the emulsion was then incubated at 80 °C for 30 min and centrifuged again at 950× *g* for 5 min. The ES was calculated using the following equation:

(8) $$ES\left(\%\right)=\frac{V_{t}}{V_{f}}\cdot 100$$

where V_t_ is the volume of the emulsified layer obtained after incubation and centrifugation.

#### 2.5.4. Total Polyphenol Content

The total polyphenol content in both pectin-rich fractions was determined by the Folin–Ciocalteu method. Briefly, 100 µL of a 2% sample solution in water was mixed with 500 µL Folin–Ciocalteu reagent, 7.4 mL of water and 2 mL of 15% (*w*/*v*) sodium carbonate. The mixture was incubated for 2 h in the dark, and the absorbance was measured at 750 nm against a blank, which contained a corresponding amount of water instead of the pectin-rich fraction solution. The results were expressed as mg GAEs (gallic acid equivalents) per g of sample based on the previously determined slope for the known concentrations of gallic acid as standard.

#### 2.5.5. Antioxidant Activity

The antioxidant activity of pectin-rich fractions was measured by the ferric-reducing antioxidant power (FRAP) method. A total of 100 µL of a 2% sample solution in water was mixed with 3 mL of FRAP reagent (mixture of sodium acetate buffer (pH 3.6), 10 mM TPTZ solution in 40 mM hydrochloric acid, and 20 mM ferric chloride hexahydrate solution in a 10:1:1 (*v*/*v*/*v*) ratio, respectively) and incubated in the dark for 5 min. The absorbance was measured at 593 nm against blank containing only the FRAP reagent. The results were expressed as µmol TE (trolox equivalents) per gram of the sample.

### 2.6. Prebiotic Activity Determination

The effect of both pectin-rich fractions on the growth of four Gram-positive bacterial strains, *S. epidermidis* DSM 20044, *S. epidermidis* ATCC 12228, *S. aureus* MRSA, and *S. aureus* ATCC 25923, was examined. The bacterial inoculums of both strains were prepared by transferring several colonies to the Brain Heart Infusion broth and allowing them to grow overnight at 37 °C.

The effect of different concentrations of CAP and AOPP solution prepared in Mueller Hinton broth was monitored by optical density (OD) measurement at 600 nm using a microplate reader (BioTek^®^ Epoch, Agilent Technologies, Santa Clara, CA, USA). Briefly, the 100 µL of corresponding pectin-rich fraction solution (0.4%) was added in duplicate to the microtiter plate, and a dilution series was prepared in Mueller Hinton (MH) medium. Each well containing 100 µL of sample received 100 µL of diluted inoculum, resulting in a final volume of 200 µL per well. The final concentration of each sample was in the range of 0.0625–2 mg/mL. Negative controls contained only MH medium, while positive controls included MH broth and inoculum. OD600 values presented in figures are obtained when the initial OD600 values (at 0 h) were subtracted from the value measured at a particular time point. The obtained OD600 values after 24 h of bacterial growth were used to calculate changes in bacterial growth relative to the control using the following equation:

(9) $$Stimulation\;or\;Inhibition\;(\%)=\frac{\left|{OD}_{sample}-{OD}_{control}\right|}{{OD}_{control}}\cdot 100$$

Prebiotic capacity (PC) was calculated as follows:

(10) $$PC=\frac{SEP24}{SEC24}-\frac{SAP24}{SAC24}$$

where SEP24 and SAP24 are OD600 values obtained when the initial OD600 values (at 0 h) were subtracted from the value measured for *S. epidermidis* and *S. aureus*, respectively, after 24 h of growth in medium with pectin-rich fraction supplementation. SEC24 and SAC24 are OD600 values obtained when the initial OD600 values (at 0 h) were subtracted from the value measured for *S. epidermidis* and *S. aureus*, respectively, after 24 h of growth in medium without pectin-rich fraction supplementation (control samples).

### 2.7. Statistical Analysis

All experiments were performed with three independent samples, with the exception of prebiotic activity determination experiments, which were performed in duplicates, and all results were presented as mean ± standard deviation from the replicates. To examine the statistical significance of mean value differences for multiple comparisons of the results (ANOVA (a one-way analysis of variance) followed by a post hoc Tuckey’s test), OriginPro 8.5 software (OriginLab Corporation, Northampton, MA, USA) was utilised. Differences at *p* ≤ 0.05 were regarded as significant. For the statistical analysis of the growth curves results, a Student’s *t*-test was applied using Microsoft Excel 2013 software (Microsoft Corporation, Redmond, WA, USA).

## 3. Results

### 3.1. Pectin-Rich Fraction Extraction and Determination of Physicochemical Properties

The overall pectin-rich fraction yield was 4.67%, whereas CAP accounted for 2.90%, and AOPP was obtained in a yield of 1.77%. These results are in accordance with previously reported yields of pectin-rich fractions from blackcurrant pomaces, which were between 2 and 15% [20,22,28]. It should be noted that different extraction methods and blackcurrant cultivars, as well as pre-processing steps, were used in all these studies; therefore, a wide range of obtained yields, as well as resulting pectin purity and structural and techno-functional properties, are expected. Table 1 further shows the pH, moisture content, a_w_, and protein concentration of extracted pectin-rich fractions since these parameters strongly affect technological features such as shelf life and emulsifying properties. CAP and AOPP solutions had pH values of 2.95 and 3.15, respectively, which indicates the anionic nature of both pectin-rich fractions. Statistical analysis showed that there were no statistically significant differences in pH values between two pectin-rich fractions. Besides being utilised as a gel-forming agent, pectin recently gained attention for being used as an emulsifier and emulsion stabiliser [29]. Although the exact correlation between the pectin structure and its emulsifying properties is not completely unrevealed, it is known that the HG:RGI ratio plays a major role in emulsion-stabilizing properties, while attached hydrophobic protein moieties, feruloyl, and acetyl residues are crucial for its emulsifying activity [29,30]. Therefore, the protein content of two pectin-rich fractions was determined, and concentrations of 1.64 mg/g and 4.97 mg/g were measured for CAP and AOPP, respectively. Both values were in accordance with previously reported ones for pectin obtained using various sources and extraction methods [18,25,31]. The obtained results indicate that AOPP has three times higher protein concentrations compared to CAP, suggesting its higher applicability in emulsion preparation. Water activities of two pectin-rich fractions were similar (0.32 for CAP and 0.34 for AOPP), as well as moisture contents (6.35% for CAP and 7.12% for AOPP), and there was no statistically significant difference between the two samples regarding these parameters. These low values are suitable for applicability in skin care products since such ingredients are less susceptible to microbiological contamination and, therefore, they have longer shelf life [25].

### 3.2. Pectin-Rich Fractions Monomeric Composition

In order to determine the monomeric composition of extracted CAP and AOPP fractions, complete acid hydrolysis was performed, and GC-FID results obtained after derivatisation are presented in Table 2, while representative chromatograms are available in the Supplementary Materials (Figure S2). As can be seen, GalA was the most abundant monosaccharide in both pectin-rich fractions, but with significantly different shares. Acid-soluble pectin obtained with citric acid (CAP) had a very high GalA content of 72.3%. On the other hand, Ca-bound pectic polysaccharides obtained using ammonium oxalate (AOPP) had a significantly lower GalA content of 48.3%, placing it among pectin-like substances. The obtained results suggest differences in both their HG content and purity. For example, when sequential extraction of acid-soluble (by HCl) and Ca-bound (by ammonium oxalate) pectin from blackcurrant pomaces from the UK and Poland was performed, a less pronounced difference between two fractions was observed, with the GalA content ranging from 70 to 80% [20]. The observed trends could indicate a strong influence of other parameters aside from the extraction method, such as fruit variety and maturity and pre-processing steps. The presence of Rha, Gal, and Ara indicated the presence of RGI subunits in the pectin structure. A lower Ara content was determined in AOPP (2.6%) than in CAP (10.4%), while AOPP was enriched in Rha units (11.4% and 3.8% for AOPP and CAP, respectively), suggesting higher content of RGI compared to CAP [18]. Interestingly, CAP had higher Gal content characteristics for RGI side chains. Low amounts (up to 1%) of Xyl were identified in both fractions, with no statistically significant differences between them. This pentose may be part of pectin, constituting the xylogalacturonan domain, or may be related to the presence of xyloglucan [32,33,34]. Regarding Glc and Man, which are indicative of the other polysaccharides such as cellulose and hemicellulose or mannans, very low amounts were detected in CAP (0.2% of Man and 1.7% of Glc). On the other hand, AOPP was moderately rich in Glc (4.4%) and had a significantly higher Man concentration (25.6%), implying a considerable mannans content. This pectic polysaccharide was probably rich in mannose because the pomace was rich in seeds, and Hilz et al. found mannose to be the dominant sugar in blackcurrant seeds [35]. Also, Kosmala et al. found a large amount of mannose after sequential polysaccharide extraction, but they did not use any acid as an extracting agent [36].

Based on monosaccharide composition, the structural parameters of CAP and AOPP were calculated (Table 3). As shown, HG was the predominant component of CAP with a 68.5% share, while AOPP had a HG content of only 36.9%. On the other hand, AOPP had a somewhat higher RGI content (32.1% compared to 28.7% in CAP). Both degrees (DB-RGI) and extents (EB-RGI) of branching were higher for CAP, supporting the presence of arabinogalactan, galactan, and/or arabinan branches attached to the RGI [25]. Pectin linearity (LP) was relatively low for both pectin-rich fractions, supporting the significant presence of RGI side branches, which was previously demonstrated to be responsible for RGI-enriched pectin’s ability to form dense gels under both cation- and acid-induced conditions [37]. Pectin purity (PP), which shows the ratio between pectin and other non-pectic polymers, was calculated, which confirmed the very high purity of CAP (50.3 times more pectin than other polymers) and significantly lower purity of AOPP (2.3 times more pectin than other polymers).

### 3.3. Molecular Weight Distribution

**Figure 2:**
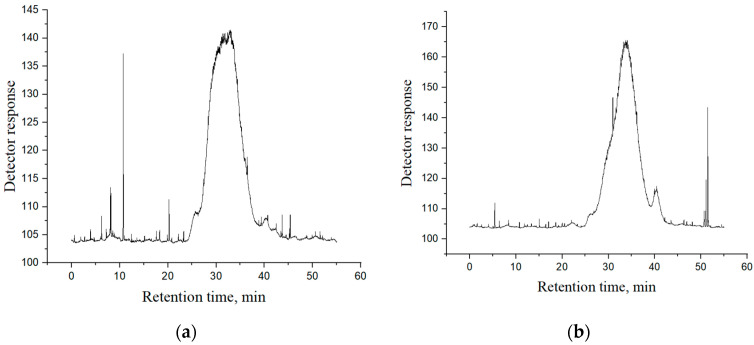
HPSEC-ELSD chromatograms of acid-soluble (CAP, (**a**)) and Ca-bound (AOPP, (**b**)) pectin-rich fractions.

Pectin gelling, thickening, and stabilising performances are dependent on its Mw. Therefore, HPSEC analysis of CAP and AOPP was performed (Figure 2). Both fractions had four fractions but with different Mw and distribution. Very high Mw fragments above 5000 kDa and 4000 kDa were present in CAP and AOPP, respectively, but they were represented in a low percentage (7.5% in CAP and 4.7% in AOPP), which could be a consequence of the aggregation of a small number of polysaccharide molecules during the lyophilisation process. AOPP had another high Mw fragment with an average Mw of 788 kDa, which accounted for 18.9%. Both fractions had one dominant peak with an average Mw of 218.1 kDa for CAP (79.8%) and 134.1 kDa for AOPP (64.2%). The lower average Mw of the dominant fragment in Ca-bound pectic polysaccharides compared to the acid-soluble pectin fraction is in accordance with previously published results for pectin obtained from blackcurrant pomaces from the UK and Poland [20]. However, it should be noted that Mw of pectin obtained in the same study were between 45.5 kDa and 109.6 kDa, which is lower compared to our results. Therefore, other factors besides the extraction method, such as fruit cultivar and maturity, climate, and growth conditions, as well as previous processing steps, could significantly influence the molecular characteristics of pectin [21,38]. Among other factors, gelling ability is dependent on pectin viscosity, which is in correlation with its molecular weight [39]. Namely, if the molecular weight is higher, the viscosity is higher, so a relatively high molecular weight of dominant portions in two samples, compared to previously isolated blackcurrant pomace pectin, could be considered favourable. Regarding low Mw fragments, CAP had two peaks corresponding to an average Mw of 7.2 kDa (8.4%) and 3.3 kDa (4.3%), while AOPP had one peak representing a fraction with an average Mw of 8.2 kDa (12.2%). Since the abundance of these low Mw fragments was relatively low, it can be assumed that the degradation of pectin molecules during extraction was not significant [25].

### 3.4. XRD Analysis

**Figure 3:**
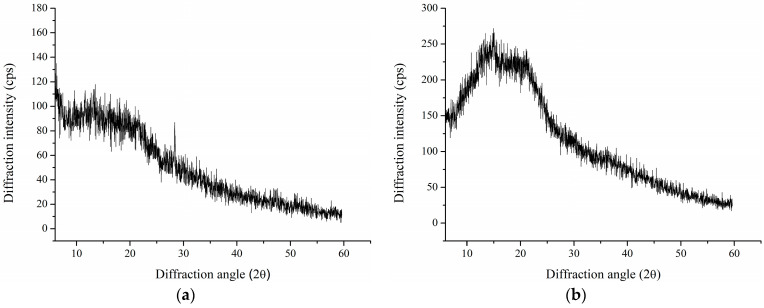
XRD spectra of (**a**) acid-soluble (CAP) and (**b**) Ca-bound (AOPP) pectin-rich fractions from blackcurrant pomace.

XRD analysis is a valuable tool for determining whether the material is amorphous or crystalline. Therefore, it was conducted for CAP and AOPP samples (Figure 3). The absence of sharp peaks in both pectin-rich fractions suggests a predominantly amorphous structure [40]. Broad humps with low intensities present in XRD patterns of both spectra could be associated with a certain amount of smaller crystals, but they can also be caused by the abundant presence of lattice defects, confirming the amorphous structure of CAP and AOPP from blackcurrant pomace [41]. Although a certain degree of crystallinity was previously reported for various pectin samples, it is generally known that amorphous solids have higher solubility and dissolution rates due to higher free energy compared to corresponding crystals [42,43,44]. It is known that dissolution is a process that involves two parallel phenomena – penetration of solvent and dissolution of polymer. It was hypothesised that crystals could make it difficult for solvent to penetrate, leading to significantly lower solubility of crystalline solid materials. Based on this, the amorphous structure of obtained pectin-rich fractions could contribute to their applicability in cosmetic formulations at a wider concentration range and lower reliance on potentially harmful solubilisers [45]. It should be noted that somewhat stronger diffraction intensity was observed for AOPP. Although crystallinity is dependent on moisture content, there were no statistically significant differences between CAP and AOPP samples (Table 1); therefore, differences in XRD profiles could not be correlated to this parameter. However, AOPP possesses a significantly higher Man content (Table 2) and stronger diffraction intensity could be associated with certain crystallinity originating from mannan polymers [46]. Based on these results, it can be assumed that CAP would have an increased water intake efficiency compared to AOPP [47].

### 3.5. FTIR Analysis

**Figure 4:**
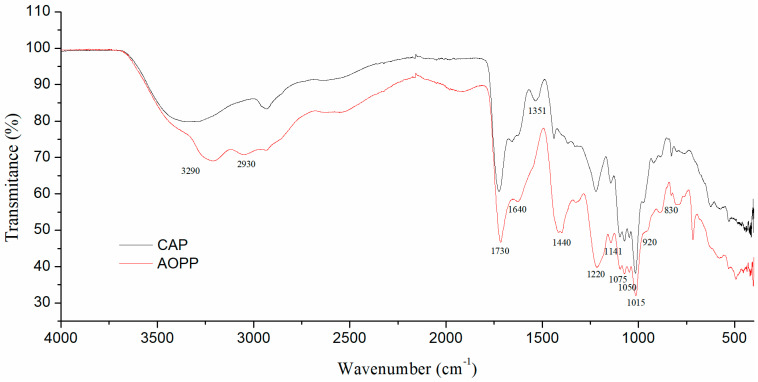
FTIR spectra of blackcurrant pomace acid-soluble (CAP) and Ca-bound (AOPP) pectin-rich fractions.

In order to prove the presence of functional groups characteristic of pectin structure, FTIR analysis of CAP and AOPP was performed (Figure 4). Both samples had major absorption peaks at 3290 cm^−1^, which are bands related to stretching the hydroxyl groups, while bands at 2930 cm^−1^ are caused by C-H stretching vibrations of CH_2_ groups [48]. These bands are typically present in pectin samples. However, it should be noted that both bands could, besides pectin, originate from bound polyphenols, which may be present in tested samples, contributing to their antioxidant activity [49]. Furthermore, the band at 1730 cm^−1^ corresponds to the methyl-esterified carboxyl group (COO-R), while the near bond at 1640 cm^−1^ comes from the stretching vibration of the free carboxyl group (COO^−^). Based on the areas of these two peaks, DM values were estimated for both pectin-rich fractions. As can be seen from Table 3, the DM value for CAP was 48.70%, while for the AOPP, it was 51.26%, placing both of them in between low- and high-methoxyl pectin. These values are very similar to results obtained by Mierczyńska et al.: 49.2% DM for blackcurrant pectin [50]. In another study, different values of DM were reported for blackcurrant pomaces with varying values (between 11% and 38%) depending on the extraction method used and the pomace source [20]. Interestingly, in the current study, there was no statistically significant difference in DM value between two pectin-rich fractions despite the fact that different methods were used for their isolation. The obtained DM values, which are in between low- and high-methoxyl pectin, indicate that the obtained fractions could potentially form gels in addition to Ca^2+^ ions (as low-methoxyl pectin) or sugars at acidic conditions (as high-methoxyl pectin), which should be subject of detailed gelation study in the future [5]. The stretching band at 1440 cm^−1^ also comes from carboxylate groups, indicating the presence of aliphatic or aromatic methyl, methylene, and methoxyl groups on the structure. Peaks at 1220 cm^−1^ and 1141 cm^−1^ originate from stretching vibrations of ether R-O-R and C-C bonds in cyclic pectin structure. Bands centred at 1075 cm^−1^ and 1050 cm^−1^ could be attributed to the presence of galactan and arabinan in the structure [25]. The band positioned at 1015 cm^−1^ could be related to glycosidic C-O linkages, which are typical for pectin backbone vibrations [51]. Peaks at 1351 cm^−1^ and 920 cm^−1^ represent the “scissoring” and “rocking” modes of the –CH_3_ group, while the band at 830 cm^−1^ originates from the deformation of the -COOH groups out of the plane [52]. In the end, bands of medium intensity below 900 cm^−1^ could be mostly attributed to C-O-C bridge vibrations, which is common for polysaccharides pyranose rings [48].

### 3.6. Techno-Functional and Antioxidant Properties

In order to evaluate the CAP and AOP of defined physicochemical characteristics as perspective cosmetic ingredients, crucial techno-functional and bioactive properties were determined (Table 4). WRC represents the ability to retain water during exposure to pressure, heating, centrifugation, or force, which strongly contribute to the texture of the product. As can be seen from Table 4, the WRC was slightly higher for CAP in comparison with AOPP, probably due to differences in chemical structures. Since the WRC is a critical property in the gel matrix, which is in positive correlation with the GalA content and HG domain of pectin, it can be assumed that CAP had a higher WRC compared to AOPP due to a higher HG proportion and higher GalA content [4,53]. Additionally, the average molecular weight of the dominant CAP fraction, calculated based on HPSEC-ELSD chromatograms, was higher compared to the molecular weight of the dominant AOPP fraction, which could also contribute to a higher WRC [5]. Furthermore, XRD analysis implied a somewhat higher crystallinity of AOPP, which may result in decreased WRC values [46]. Regarding the ability of the two pectin-rich fractions to hold oil, which is usually an indicator of good emulsifying properties, there was no statistically significant difference between them, and values were higher than data in the literature for other berries’ pectic polysaccharides, which may be caused by higher amounts of high Mw molecules [18]. In order to further examine extracted pectin-rich fractions as emulsifiers for future application in cosmetic products, their emulsifying capacity and emulsifying stability were determined. As shown in Table 4, AOPP had a significantly higher EC (49.3% versus 33.3% for CAP), which could be a consequence of previously determined higher protein content (Section 3.1). Namely, hydrophobic protein moieties can be adsorbed at the oil–water interface and form stabilising layers that surround oil droplets [53]. The EC value obtained for CAP is in accordance with values previously reported for pectin isolated from different berry pomaces by various extraction methods, while the AOPP fraction had a significantly higher EC [25]. Although these values are lower compared to commercial pectin, it should be noted that emulsion stability is another important parameter. Both emulsions had excellent stabilities with ES values of 100.0% and 94.1% for CAP and AOPP, respectively, which were significantly higher than previously reported for commercial pectin and pectin from other sources [27,54].

Besides protein, berry pectin is known to be rich in bound polyphenols, which contribute to their multi-functionality in topical formulations since they provide antioxidant, anti-inflammatory, and UV-protective effects. As can be seen from Table 4, CAP had a total phenolic content (TPC) of 43.80 mg GAE/g, while AOPP had a TPC of 4.59 mg GAE/g. Higher polyphenol content in CAP could be, at least to some extent, associated with a higher Ara share (Table 2) originating from arabinan side chains, which could be responsible for interactions with polyphenols [55]. However, this ten-fold higher value of TPC for CAP was not accompanied by the same trend in antioxidant activity, which was only three times higher compared to AOPP, indicating that part of the antioxidant activity could be originating from pectic polysaccharides themselves [56].

### 3.7. Prebiotic Activity

As the final part of the current study, the influence of CAP and AOPP extracted from blackcurrant pomace on two skin bacteria was examined. Although the concept of skin probiotics and prebiotics is still evolving, a number of studies suggest *S. epidermidis* as a perspective “next generation” skin probiotic [57]. On the other hand, *S. aureus* is the most common skin pathogen, with approximately 90% of atopic dermatitis patients suffering from infection caused by its colonisation [58]. Therefore, *S. epidermidis* (DSM 2044 and ATCC 12228 strains) was chosen as a representative of beneficial skin microbiota, while *S. aureus* (ATCC 25923 and *S. aureus* MRSA strains) was selected as a pathogen. Both fractions were supplemented to the growth medium at varying concentrations and microbial growth was followed during 24 h and compared with growth in a control sample, without supplementation (corresponding *p* values for each data point presented in Tables S1 and S2).

**Figure 5:**
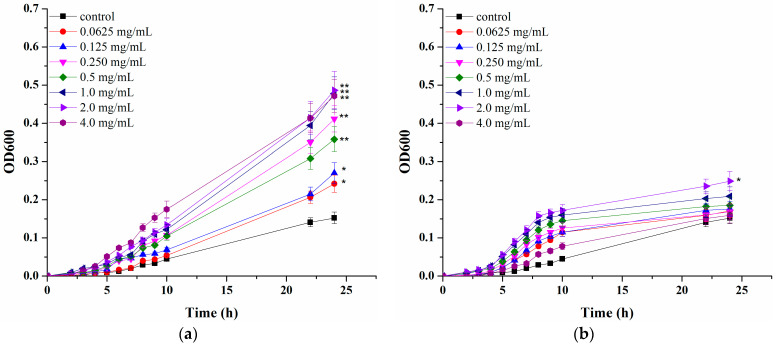

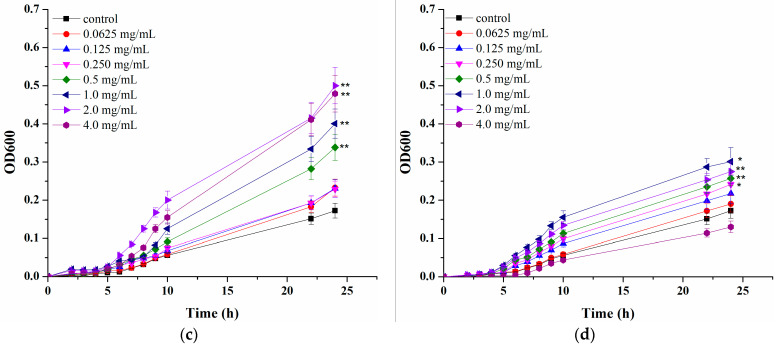
Influence of pectin-rich fractions isolated from blackcurrant pomace on *S. epidermidis* DSM 20044 ((**a**) acid-soluble and (**b**) Ca-bound) and *S. epidermidis* ATCC 12228 ((**c**) acid-soluble and (**d**) Ca-bound) strains growth. Experiments are performed in duplicates and figures represent mean values with standard deviations. Asterisk signs represent statistically significant differences between various pectin-rich fractions concentrations and the control sample (*—0.05 ≤ *p* ≤ 0.1 and **—0.01 ≤ *p* ≤ 0.05).

**Figure 6:**
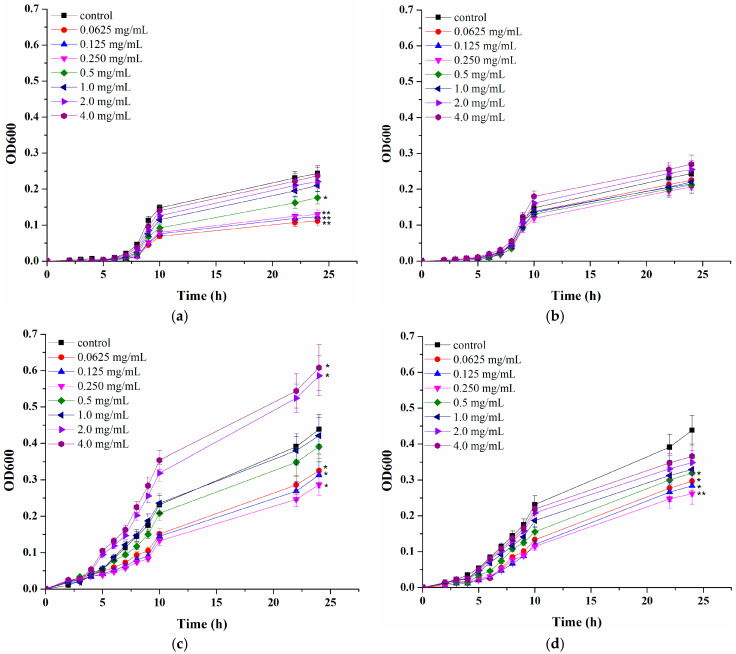
Influence of pectin-rich fractions isolated from blackcurrant pomace on *S. aureus* ATCC 25923 ((**a**) acid-soluble and (**b**) Ca-bound) and *S. aureus* MRSA ((**c**) acid-soluble and (**d**) Ca-bound) strain growth. Experiments are performed in duplicates and figures represent mean values with standard deviations. Asterisk signs represent statistically significant differences between various pectin-rich fractions concentrations and the control sample (*—0.05 ≤ *p* ≤ 0.1 and **—0.01 ≤ *p* ≤ 0.05).

It can be noticed that both pectin-rich fractions had a more pronounced effect on shortening the lag phase and increasing the specific microbial growth rate of *S. epidermidis* DSM 20044 compared to *S. epidermidis* ATCC 12228 at certain concentrations, but this was not necessarily accompanied by higher stimulation degree after 24 h. As can be seen from Figure 5, CAP and AOPP both exhibited stimulatory effects on both *S. epidermidis* strains, but with different influences of varying concentrations and stimulation degrees. CAP demonstrated the highest stimulation degree (220%) of *S. epidermidis* DSM 20044 at 2 mg/mL, while AOPP showed the highest stimulatory effect of 64% against this strain at the same concentration. For *S. epidermidis* ATCC 12228, CAP also exhibited a significantly higher stimulatory effect (maximum of 190% at 2 mg/mL) compared to AOPP (maximum of 75% at 1 mg/mL). As can be seen from Figure 5, even an inhibitory effect is observed at high concentrations, probably due to the presence of high concentrations of certain polyphenols, which exhibited antimicrobial properties [23]. Regarding the effect of both pectin-rich fractions on *S. aureus* strain growth (Figure 6), the effect of their supplementation did not show a pronounced effect during the initial cultivation stage. However, during prolonged exposure, a significant influence was observed at certain concentrations, with the exception of AOPP and *S. aureus* ATCC 25923, where no statistically significant influence was detected at any of the tested concentrations. The highest *S. aureus* ATCC 25923 inhibition degree of 54% was accomplished at 0.25 mg/mL of CAP while *S. aureus* MRSA was maximally inhibited at 0.25 mg/mL of both CAP (34%) and AOPP (40%). The inhibitory effect, which is higher at lower applied concentrations of pectic polysaccharides, is not very common; however, it should be noted that bound polyphenols could also affect microbial growth. Therefore, the overall effect is probably a result of their joint influence, both stimulatory and inhibitory. It was already proven that coagulase-negative *S. epidermidis* and coagulase-positive *S. aureus* possess different metabolic capacities for using specific nutrients, including polyphenols. We previously demonstrated that polyphenol-rich extracts of different berry fruits exhibit opposite effects on these skin bacteria at certain concentrations [23,59]. It can be assumed that, although both microorganisms are pectinase producers capable of degrading pectin, there are differences in their metabolisms, which caused different responses to examined pectin-rich fractions and bound polyphenols. In order to fully reveal mechanisms by which bacterial growth is affected, additional examinations are needed in the future.

**Figure 7:**
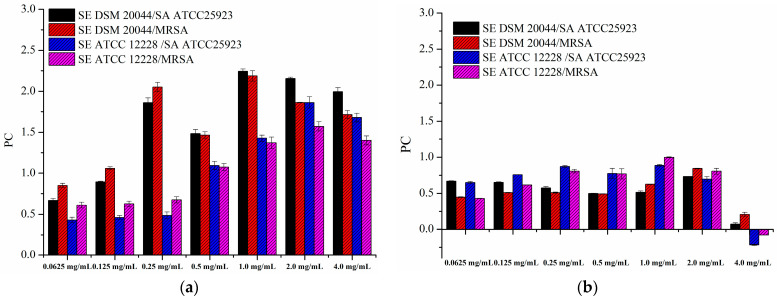
Prebiotic capacity of (**a**) acid-soluble (CAP) and (**b**) Ca-bound (AOPP) pectin-rich fractions from blackcurrant pomace. Experiments are performed in duplicates and figures represent mean values with standard deviations.

In the end, prebiotic capacity after 24 h of cultivation was calculated in order to estimate the effect of CAP and AOPP on the *S. epidermidis* and *S. aureus* ratio and select concentrations with the highest prebiotic potential against tested strains. As can be seen from Figure 7, prebiotic capacity (PC) was above zero within a very wide range, indicating a positive effect of two pectin-rich fractions on the balance of examined strains. In general, higher values were achieved for CAP (Figure 6a) compared to AOPP (Figure 6b), but it should be noted that very high stimulation of *S. epidermidis* strains significantly contributed to this result, even at concentrations at which certain *S. aureus* stimulation was observed (2 mg/mL and 4 mg/mL of CAP). Therefore, lower concentrations (0.25–1 mg/mL of CAP and 0.0625–2 mg/mL of AOPP) that exhibited the desired effect on all individual strains and still gave high PC values should be selected as optimum. For the comparison, when we previously examined the influence of blackcurrant pomace polyphenols on the growth of *S. epidermidis* DSM 20044 and *S. aureus* ATCC 25923, the highest PC values of around 0.5 were reached, indicating that AOPP and CAP are both promising skin prebiotics [59]. Finally, it should be noted that, although complete inhibition of *S. aureus* strains was not achieved, a simultaneous stimulatory effect on *S. epidermidis* strains is expected to enhance the natural defence mechanisms of the skin by additionally combating *S. aureus* colonization in a symbiotic manner [1]. Moreover, cosmetic products with incorporated CAP or AOPP from blackcurrant pomace could be potentially useful as a complementary treatment during and after systemic or topical antibiotic therapy, since they could support the repopulation of the skin by beneficial *S. epidermidis* and speed up the skin microbiota rebalancing process.

## 4. Conclusions

The current study demonstrated that blackcurrant pomace left after juice extraction is a suitable raw material for sequential extraction of acid-soluble and Ca-bound pectin-rich fractions. Although acid-soluble pectin obtained using citric acid was of significantly higher purity, both fractions demonstrated very promising techno-functional and bioactive properties important for application in cosmetics. Since both pectin-rich fractions stimulated beneficial *S. epidermidis* and inhibited harmful *S. aureus* growth they should be further examined as prospective skin prebiotics. The overall results suggest that this innovative and appealing approach for the valorisation of blackcurrant pomace derived from the production of juice could be of great importance for obtaining a novel class of multifunctional cosmetic ingredients. Further optimization of the pectin isolation procedure and studying other properties should be performed in the future, including comprehensive characterization of two fractions and prebiotic activity testing using more complex samples of skin microbiota.

## Figures and Tables

**Table 1:** Extraction yields and main physicochemical properties of acid-soluble (CAP) and Ca-bound (AOPP) pectin-rich fractions from blackcurrant pomace.

Sample	Yield (% (*w*/*w*))	pH	Moisture (%)	a_w_	Proteins (mg/g)
CAP	2.90 ± 0.18 ^a^	2.95 ± 0.14 ^a^	6.35 ± 0.54 ^a^	0.32 ± 0.01 ^a^	1.64 ± 0.10 ^b^
AOPP	1.77 ± 0.17 ^b^	3.15 ± 0.18 ^a^	7.12 ± 0.63 ^a^	0.34 ± 0.02 ^a^	4.97 ± 0.27 ^a^

Different characters in the same column signify a statistically significant difference between samples at *p* ≤ 0.05. Experiments were conducted in triplicate.

**Table 2:** Monosaccharide composition (%) of acid-soluble (CAP) and Ca-bound (AOPP) pectin-rich fractions from blackcurrant pomace.

Sample	Xyl	Ara	Rha	Gal	Man	Glc	GalA
CAP	0.86 ± 0.05 ^a^	10.44 ± 0.80 ^a^	3.81 ± 0.23 ^b^	10.63 ± 0.36 ^a^	0.24 ± 0.02 ^b^	1.69 ± 0.08 ^b^	72.32 ± 6.41 ^a^
AOPP	0.93 ± 0.04 ^a^	2.63 ± 0.15 ^b^	11.36 ± 0.67 ^a^	6.76 ± 0.32 ^b^	25.62 ± 1.49 ^a^	4.40 ± 0.28 ^a^	48.30 ± 3.68 ^b^

Different characters in the same column signify a statistically significant difference between samples at *p* ≤ 0.05. Experiments were conducted in triplicate.

**Table 3:** Structural parameters based on the monomeric composition of acid-soluble (CAP) and Ca-bound (AOPP) pectin-rich fractions from blackcurrant pomace.

Sample	HG (%)	RGI (%)	DB-RGI	EB-RGI	LP	PP	DM (%)
CAP	68.52 ± 2.36 ^a^	28.69 ± 1.44 ^b^	18.99 ± 1.36 ^a^	5.54 ± 0.20 ^a^	2.91 ± 0.06 ^a^	50.26 ± 1.87 ^a^	48.70 ± 3.15 ^a^
AOPP	36.94 ± 1.09 ^b^	32.11 ± 1.01 ^a^	4.25 ± 0.18 ^b^	0.83 ± 0.04 ^b^	2.33 ± 0.09 ^b^	2.30 ± 0.18 ^b^	51.26 ± 4.61 ^a^

Different characters in the same column signify a statistically significant difference between samples at *p* ≤ 0.05. Experiments were conducted in triplicate.

**Table 4:** Techno-functional and bioactive properties of blackcurrant pomace acid-soluble (CAP) and (B) Ca-bound (AOPP) pectin-rich fractions.

Sample	WRC (mL/g)	OHC (mL/g)	EC (%)	ES (%)	TPC (mg GAE/g)	FRAP (µmol TE/g)
CAP	9.97 ± 0.42 ^a^	18.96 ± 1.27 ^a^	33.33 ± 2.42 ^b^	100.00 ± 0.03 ^a^	43.80 ± 3.24 ^a^	131.42 ± 5.47 ^a^
AOPP	7.32 ± 0.50 ^b^	19.32 ± 1.39 ^a^	49.33 ± 3.07 ^a^	94.12 ± 3.48 ^b^	4.59 ± 0.37 ^b^	45.21 ± 2.19 ^b^

Different characters in the same column signify a statistically significant difference between samples at *p* ≤ 0.05. Experiments were conducted in triplicate.

## Data Availability

The data presented in this study will be made openly available on a data repository immediately after publication.

## Supplementary Materials

The following supporting information can be downloaded at https://www.mdpi.com/article/10.3390/polym16192779/s1, Figure S1. Photographs of different fractions obtained during pectin isolation; Figure S2. Representative GC-FID chromatograms of acid-soluble (CAP, a) and Ca-bound (AOPP, b) pectin-rich fractions monosaccharide composition obtained after complete hydrolysis. Table S1. Results of a two-tailed Student’s *t*-test of acid-soluble (CAP) and Ca-bound (AOPP) pectin-rich fractions influence on *S. epidermidis* strains growth compared to control sample without supplementation. Table S2. Results of a two-tailed Student’s *t*-test of acid-soluble (CAP) and Ca-bound (AOPP) pectin-rich fractions influence on *S. aureus* strains growth compared to control sample without supplementation.

## Abbreviations

CAP	Citric acid pectin
AOPP	Ammonium oxalate pectic polysaccharides
Mw	Molecular weight
HG	Homogalacturonan
RGI	Rhamnogalacturonan I
DM	Degree of methyl esterification
TPC	Total phenolic content
WRC	Water retention capacity
OHC	Oil holding capacity
DB-RGI	Degree of branching of rhamnogalacturonan I
EB-RGI	Extent of branching of rhamnogalacturonan I
LP	Linearity of pectin
PP	Pectin purity
GalA	Galacturonic acid
Rha	Rhamnose
Xyl	Xylose
Ara	Arabinose
Gal	Galactose
Glc	Glucose
Man	Mannose
GC-FID	Gas Chromatography coupled to Flame Ionisation Detector
HPSEC-ELSD	High-Performance Size-Exclusion Chromatography (HPSEC) coupled to Evaporative Light Scattering Detector (ELSD)
FTIR	Fourier Transform Infrared spectroscopy
XRD	X-ray Diffraction
