# Supplementary material for: Valorisation of Blackcurrant Pomace by Extraction of Pectin-Rich Fractions: Structural Characterization and Evaluation as Multifunctional Cosmetic Ingredient
Source: Polymers (Basel). 2024 Sep 30;16(19):2779. doi: 10.3390/polym16192779 (PMC11478554; doi:10.3390/polym16192779)
Supplement: Supplementary file 1 [file polymers-16-02779-s001.zip › polymers-3160466-supplementary.pdf]

Supplementary material of the manuscript

Valorisation of blackcurrant pomace by extraction of pectin rich fractions – structural characterization and evaluation as multifunctional cosmetic ingredient

Marija Ćorović[1*]

Anja Petrov Ivanković[2]

Ana Milivojević[1]

Milica Veljković[2]

Milica Simović[1]

Paula Lopez-Revenga[3]

Antonia Montilla[3]

F. Javier Moreno[3]

Dejan Bezbradica[1]

[1]Faculty of Technology and Metallurgy, University of Belgrade, Karnegijeva 4, 11000 Belgrade

[2]Innovation Center of Faculty of Technology and Metallurgy, Karnegijeva 4, 11000 Belgrade, Serbia

[3] Grupo de Química y Funcionalidad de Carbohidratos y Derivados, Instituto de Investigación en Ciencias de la Alimentación, CIAL (CSIC-UAM), Madrid, Spain

[*] Corresponding author: Marija Ćorović, Department of Biochemical Engineering and Biotechnology, Faculty of Technology and Metallurgy, University of Belgrade, Karnegijeva 4, 11000 Belgrade, Serbia. E-mail address: mstojanovic@tmf.bg.ac.rs Tel.: (+38111) 3303727. Fax: (+38111) 3370387.

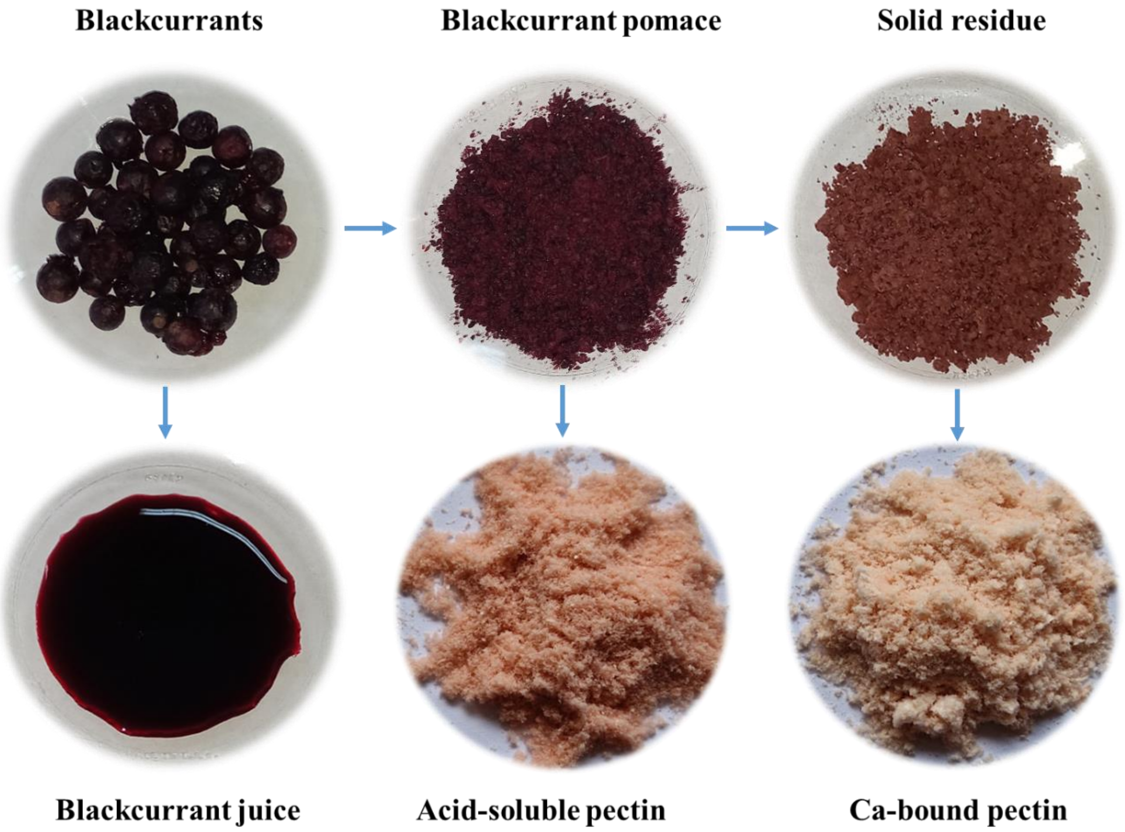

Figure S1. Photographs of different fractions obtained during pectin rich fractions isolation.

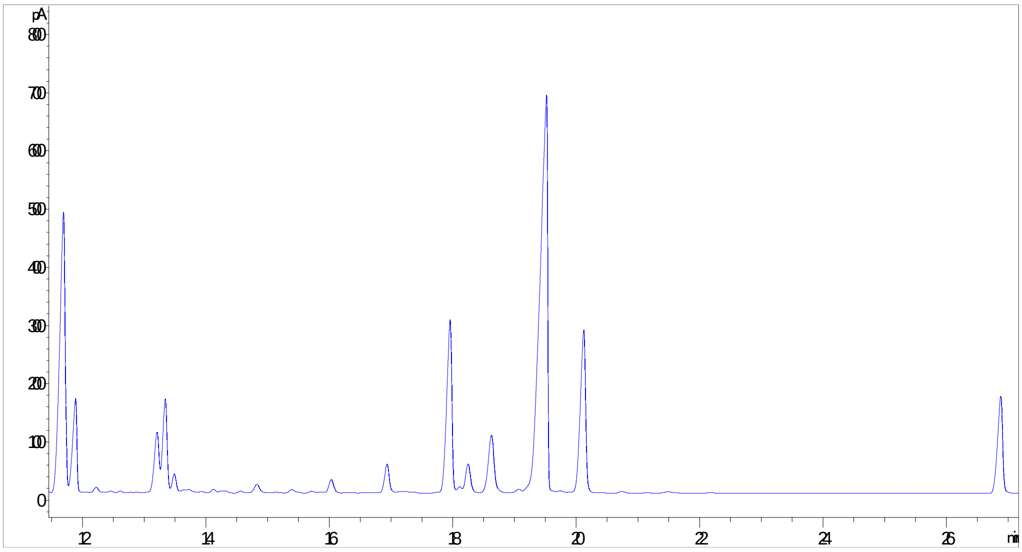

(a)

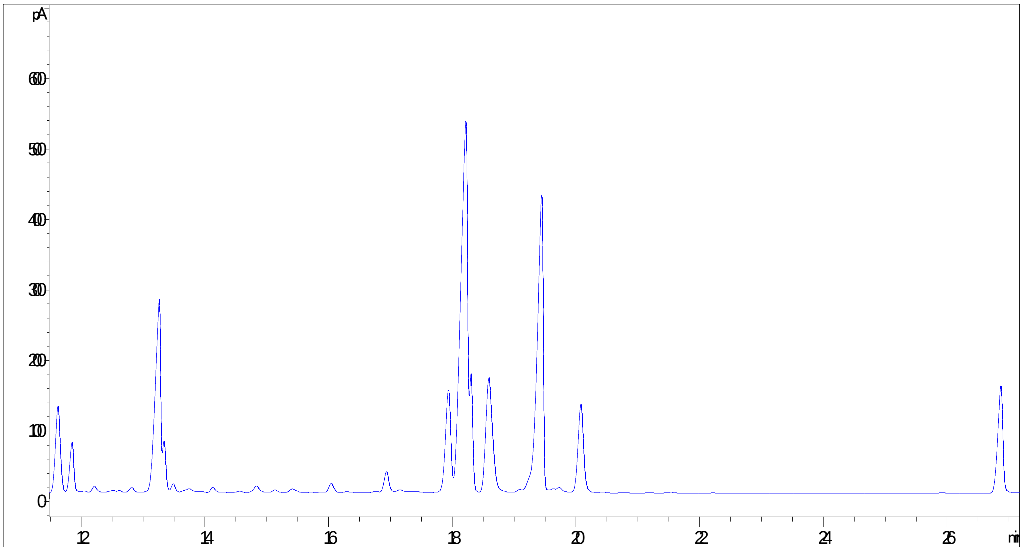

(b)

Figure S2. Representative GC-FID chromatograms of acid soluble (CAP, a) and Ca-bound (AOP, b) pectin rich fractions monosaccharide composition obtained after complete hydrolysis.

Table S1. Results of a two-tailed Student's t-test of acid soluble (CAP) and Ca-bound (AOP) pectin rich fractions influence on *S. epidermidis* strains growth compared to control sample without supplementation.

a) P values for *S. epidermidis* DSM 20044 and CAP							
**Time**	**0.0625 mg /mL**	**0.125 mg/mL**	**0.25 mg/mL**	**0.5 mg/mL**	**1 mg/mL**	**2 mg/mL**	**4 mg/mL**
**2 h**	0.015	0.028	0.013	0.027	0.019	0.024	0.030
**3 h**	0.016	0.026	0.015	0.027	0.032	0.038	0.044
**4 h**	0.200	0.096	0.018	0.024	0.017	0.025	0.035
**5 h**	0.254	0.030	0.010	0.023	0.014	0.023	0.032
**6 h**	0.039	0.023	0.014	0.026	0.016	0.025	0.033
**7 h**	0.557	0.019	0.009	0.020	0.021	0.032	0.033
**8 h**	0.044	0.030	0.013	0.024	0.026	0.035	0.036
**9 h**	0.061	0.034	0.014	0.025	0.028	0.036	0.037
**10 h**	0.179	0.071	0.048	0.055	0.060	0.067	0.065
**22 h**	0.049	0.051	0.037	0.045	0.045	0.054	0.045
**24 h**	0.053	0.054	0.031	0.037	0.038	0.047	0.038

b) P values for *S. epidermidis* DSM 20044 and AOP							
**Time**	**0.0625 mg /mL**	**0.125 mg/mL**	**0.25 mg/mL**	**0.5 mg/mL**	**1 mg/mL**	**2 mg/mL**	**4 mg/mL**
**2 h**	0.035	0.042	0.046	0.041	0.042	0.033	0.027
**3 h**	0.037	0.042	0.045	0.043	0.043	0.035	0.028
**4 h**	0.287	0.543	0.064	0.052	0.049	0.037	0.234
**5 h**	0.046	0.050	0.051	0.052	0.050	0.042	0.052
**6 h**	0.048	0.051	0.057	0.053	0.052	0.044	0.056
**7 h**	0.053	0.056	0.061	0.056	0.055	0.047	0.062
**8 h**	0.036	0.040	0.048	0.045	0.045	0.036	0.068
**9 h**	0.019	0.024	0.032	0.032	0.032	0.023	0.059
**10 h**	0.042	0.046	0.054	0.052	0.052	0.042	0.076
**22 h**	0.332	0.197	0.345	0.161	0.059	0.037	0.543
**24 h**	0.384	0.316	0.467	0.227	0.130	0.061	0.745

c) P values for *S. epidermidis* ATCC 12228 and CAP							
**Time**	**0.0625 mg /mL**	**0.125 mg/mL**	**0.25 mg/mL**	**0.5 mg/mL**	**1 mg/mL**	**2 mg/mL**	**4 mg/mL**
**2 h**	0.029	0.029	0.016	0.024	0.021	0.020	0.033
**3 h**	0.019	0.040	0.024	0.029	0.034	0.034	0.049
**4 h**	0.713	0.035	0.051	0.081	0.015	0.016	0.059
**5 h**	0.043	0.035	0.019	0.026	0.011	0.010	0.031
**6 h**	0.064	0.022	0.018	0.026	0.014	0.018	0.032
**7 h**	0.923	0.024	0.030	0.024	0.020	0.024	0.033
**8 h**	0.615	0.049	0.033	0.031	0.030	0.028	0.037
**9 h**	0.818	0.214	0.293	0.044	0.028	0.028	0.039
**10 h**	0.735	0.223	0.128	0.079	0.062	0.059	0.056
**22 h**	0.167	0.136	0.132	0.053	0.045	0.045	0.049
**24 h**	0.104	0.116	0.109	0.044	0.037	0.038	0.042

d) P values for *S. epidermidis* ATCC 12228 and AOP

Time	0.0625 mg /mL	0.125 mg/mL	0.25 mg/mL	0.5 mg/mL	1 mg/mL	2 mg/mL	4 mg/mL
2 h	0.049	0.996	0.027	0.271	0.133	0.018	0.053
3 h	0.036	0.042	0.029	0.998	0.046	0.022	0.996
4 h	0.733	0.751	0.725	0.695	0.078	0.038	0.040
5 h	0.178	0.232	0.122	0.015	0.054	0.028	0.042
6 h	0.505	0.056	0.045	0.022	0.055	0.035	0.039
7 h	0.669	0.075	0.054	0.020	0.059	0.035	0.044
8 h	0.619	0.047	0.032	0.009	0.046	0.020	0.064
9 h	0.597	0.042	0.019	0.009	0.028	0.007	0.092
10 h	0.682	0.072	0.042	0.016	0.052	0.025	0.159
22 h	0.337	0.147	0.085	0.036	0.025	0.022	0.117
24 h	0.472	0.196	0.100	0.050	0.076	0.049	0.147

Table S2. Results of a two-tailed Student's t-test of acid soluble (CAP) and Ca-bound (AOP) pectin rich fractions influence on *S. aureus* strains growth compared to control sample without supplementation.

a) P values for S. aureus ATCC 25923 and CAP

Time	0.0625 mg /mL	0.125 mg/mL	0.25 mg/mL	0.5 mg/mL	1 mg/mL	2 mg/mL	4 mg/mL
2 h	0.190	0.735	0.394	0.510	0.424	0.763	0.831
3 h	0.065	0.299	0.155	0.252	0.160	0.438	0.611
4 h	0.152	0.835	0.607	0.917	0.705	0.789	0.383
5 h	0.006	0.028	0.047	0.010	0.015	0.007	0.009
6 h	0.168	0.321	0.697	0.681	0.045	0.045	0.015
7 h	0.293	0.524	0.173	0.336	0.220	0.115	0.051
8 h	0.569	0.799	0.092	0.054	0.236	0.753	0.181
9 h	0.796	0.919	0.154	0.221	0.244	0.576	0.447
10 h	0.515	0.348	0.135	0.217	0.354	0.416	0.143
22 h	0.461	0.319	0.188	0.234	0.263	0.603	0.346
24 h	0.464	0.294	0.206	0.290	0.346	0.671	0.380

b) P values for S. aureus ATCC 25923 and AOP

Time	0.0625 mg /mL	0.125 mg/mL	0.25 mg/mL	0.5 mg/mL	1 mg/mL	2 mg/mL	4 mg/mL
2 h	0.041	0.041	0.041	0.041	0.041	0.042	0.042
3 h	0.026	0.028	0.031	0.019	0.031	0.032	0.032
4 h	0.031	0.031	0.030	0.029	0.030	0.030	0.030
5 h	0.064	0.071	0.078	0.193	0.355	0.671	0.764
6 h	0.067	0.067	0.068	0.065	0.083	0.125	0.339
7 h	0.045	0.043	0.043	0.038	0.047	0.072	0.176
8 h	0.017	0.015	0.014	0.010	0.014	0.023	0.111
9 h	0.052	0.055	0.056	0.064	0.088	0.125	0.250
10 h	0.011	0.013	0.014	0.024	0.067	0.148	0.529
22 h	0.023	0.031	0.032	0.057	0.171	0.346	0.677
24 h	0.030	0.039	0.040	0.077	0.229	0.359	0.788

c) P values for S. aureus MRSA and CAP

Time	0.0625 mg /mL	0.125 mg/mL	0.25 mg/mL	0.5 mg/mL	1 mg/mL	2 mg/mL	4 mg/mL
**2 h**	0.029	0.015	0.009	0.029	0.048	0.008	0.008
**3 h**	0.077	0.206	0.118	0.044	0.182	0.218	0.073
**4 h**	0.296	0.666	0.859	0.120	0.470	0.044	0.024
**5 h**	0.272	0.095	0.076	0.836	0.684	0.026	0.019
**6 h**	0.076	0.061	0.059	0.466	0.812	0.045	0.024
**7 h**	0.054	0.041	0.051	0.156	0.560	0.084	0.052
**8 h**	0.061	0.057	0.063	0.160	0.867	0.055	0.031
**9 h**	0.050	0.044	0.045	0.233	0.565	0.047	0.042
**10 h**	0.079	0.079	0.064	0.414	0.877	0.067	0.042
**22 h**	0.090	0.070	0.061	0.376	0.803	0.071	0.075
**24 h**	0.100	0.086	0.062	0.370	0.749	0.998	0.100

d) P values for S. aureus MRSA and AOP

Time	0.0625 mg /mL	0.125 mg/mL	0.25 mg/mL	0.5 mg/mL	1 mg/mL	2 mg/mL	4 mg/mL
**2 h**	0.092	0.038	0.033	0.612	0.152	0.071	0.042
**3 h**	0.083	0.041	0.037	0.042	0.349	0.608	0.966
**4 h**	0.041	0.036	0.032	0.031	0.044	0.058	0.086
**5 h**	0.040	0.035	0.031	0.044	0.131	0.288	0.447
**6 h**	0.043	0.040	0.035	0.039	0.179	0.422	0.674
**7 h**	0.039	0.034	0.030	0.050	0.158	0.351	0.538
**8 h**	0.070	0.053	0.051	0.105	0.158	0.353	0.576
**9 h**	0.078	0.063	0.057	0.095	0.154	0.344	0.567
**10 h**	0.075	0.062	0.056	0.085	0.191	0.403	0.617
**22 h**	0.081	0.068	0.053	0.119	0.144	0.240	0.313
**24 h**	0.071	0.060	0.047	0.093	0.113	0.145	0.202
